# Generation of a high confidence set of domain–domain interface types to guide protein complex structure predictions by AlphaFold

**DOI:** 10.1093/bioinformatics/btae482

**Published:** 2024-08-22

**Authors:** Johanna Lena Geist, Chop Yan Lee, Joelle Morgan Strom, José de Jesús Naveja, Katja Luck

**Affiliations:** Institute of Molecular Biology (IMB) gGmbH, Mainz 55128, Germany; Institute of Molecular Biology (IMB) gGmbH, Mainz 55128, Germany; Institute of Molecular Biology (IMB) gGmbH, Mainz 55128, Germany; Institute of Molecular Biology (IMB) gGmbH, Mainz 55128, Germany; 3rd Medical Department, University Medical Center, Johannes Gutenberg University Mainz, Mainz 55131, Germany; University Cancer Center, University Medical Center, Johannes Gutenberg University Mainz, Mainz 55131, Germany; Institute of Molecular Biology (IMB) gGmbH, Mainz 55128, Germany

## Abstract

**Motivation:**

While the release of AlphaFold (AF) represented a breakthrough for the prediction of protein complex structures, its sensitivity, especially when using full length protein sequences, still remains limited. Modeling success rates might increase if AF predictions were guided by likely interacting protein fragments. This approach requires available sets of highly confident protein–protein interface types. Computational resources, such as 3did, infer interacting globular domain types from observed contacts in protein structures. Assessing the accuracy of these predicted interface types is difficult because we lack hand-curated reference sets of verified domain–domain interface (DDI) types.

**Results:**

To improve protein complex modeling of DDIs by AF, we manually inspected 80 randomly selected DDI types from the 3did resource to generate a first reference set of DDI types. Identified cases of DDI type nonapproval (40%) primarily resulted from inaccurate Pfam domain matches, crystal contacts, and synthetic protein constructs. Using logistic regression, we predicted a subset of 2411 out of 5724 considered DDI types in 3did to be of high confidence, which we subsequently applied to 53 000 human–protein interactions to predict DDIs followed by AF modeling. We obtained highly confident AF models for 604 out of 1129 predicted DDIs. Of note, for 47% of them no confident AF structural model could be obtained using full length protein sequences.

**Availability and implementation:**

Code is available at https://github.com/KatjaLuckLab/DDI_manuscript.

## 1 Introduction

Protein–protein interactions (PPIs) shape complex communication networks that modulate essentially all molecular pathways within the cell (Braun and Gingras 2012). A comprehensive structural and functional understanding of these molecular interaction networks is thus a prerequisite to study cellular mechanisms in health and disease (Vidal *et al.* 2011). While the systematic mapping of protein interactomes is well advanced for many model organisms, including human, experimental methods lack in throughput to provide a structural resolution of protein interactomes (Aloy *et al.* 2004, Luck *et al.* 2020, Huttlin *et al.* 2021, Michaelis *et al.* 2023). While numerous studies reported on the ability of AlphaFold and AlphaFold-Multimer (AF) to accurately predict protein complex structures of previously known and novel modes of protein binding, overall sensitivity and specificity estimates remain very modest (Akdel *et al.* 2022, Evans *et al.* 2022, Tsaban *et al.* 2022). Using small protein fragments for interface prediction by AF, we and others have reported sensitivities and specificities in the range of 65–80% and 75–80%, respectively (Bret *et al.* 2024, Lee *et al.* 2024). However, when using full-length protein sequences for AF complex structure prediction, sensitivities were reported to drop to 5% depending on the mode of protein binding (Lee *et al.* 2024). At this point, it remains largely unclear why for certain PPIs AF would fail to accurately predict a structural model using full length sequences but would succeed if given smaller protein fragments. To overcome this drop in sensitivity, we have developed and successfully applied in the past systematic protein fragmentation of interacting protein sequences and modeling of the resulting fragment pairs by AF (Lee *et al.* 2024). The drawback of this approach is a massive increase in compute time as well as a substantial increase in false discovery rate. A solution to this problem would be to guide AF predictions by selecting protein fragments that likely comprise the interaction interface. For known modes of protein binding, this can be achieved by establishing a high quality set of interface types and by performing sequence pattern searches using hidden Markov models (HMMs) or regular expressions, for example, to find instances of these interface types in two interacting protein sequences (Weatheritt *et al.* 2012, Mosca *et al.* 2013).

The largest collections of interface types are those that include globular domains binding each other (Wodak and Janin 2002, Liddington 2004). Globular domains are independent folding units in proteins with well-conserved sequences and structures. Typically, they are compact regions of around 100–250 residues with a unique tertiary fold and a hydrophobic core (Janin and Wodak 1983). Hundreds of different domain folds/types have emerged throughout the course of evolution. Occurrences of the same domain type share specific sequence signatures, which can be captured in HMMs. These HMMs can then be used to reliably predict domain occurrences in protein sequences. The most comprehensive collection of HMMs for protein domains is the Pfam database (recently merged with InterPro) (Finn *et al.* 2014, Paysan-Lafosse *et al.* 2023). In an effort to generate a collection of domain–domain interface (DDI) types, researchers used Pfam HMMs to scan protein sequences in structures deposited in the PDB to identify domain occurrences and atomic contacts between them, resulting in the 3did (3D interacting domains database) resource that currently hosts 14 972 putative DDI types (Mosca *et al.* 2014). The definition of domain–domain contacts in 3did is distance-based: A domain pair must exhibit at least five noncovalent contacts such as hydrogen bonds, salt bridges, or van der Waals interactions to be considered a possible DDI type (Stein *et al.* 2011). The accuracy of DDI type predictions using this approach strongly depends on HMMs to accurately capture folded domains and their boundaries.

Resources like DOMINE combined DDIs from 3did with DDI predictions from a variety of different tools, resulting in larger predicted DDI collections among which DDIs derived from 3did were deemed as the highest confidence (Yellaboina *et al.* 2011). Many computational studies have also used 3did content to develop more sophisticated tools to predict interfaces between proteins (Zhang *et al.* 2016, Zheng *et al.* 2021, Karan *et al.* 2022, Luther *et al.* 2023). Thus, 3did represents a very valuable, structure-based resource of inferred DDI types. However, evaluation of the accuracy of inferred DDI types is difficult because we lack reference datasets of manually curated domain-domain interactions and DDI types.

To address this need, we manually curated 80 randomly selected DDI types from 3did and evaluated that 60% of them correspond to bona fide DDI types. Nonapproval of DDI types often resulted from identified crystal contacts (28%), minimal contacts between folded domains (37%), and HMMs that do not correspond to folded domains (16%). Using the curated DDI types, we trained a logistic regression model to predict a subset of high confidence DDI types in 3did. Using this subset of DDI types and available human PPI data, we confidently predicted a DDI for 1533 PPIs. Subsequent structural modeling of 1129 of these predicted DDIs by AF resulted in highly confident models for 604 PPIs. Of note, for 47% of them, no confident AF model could be obtained using full-length protein sequences.

## 2 Materials and methods

Computer code for the processing and analysis of all data apart from the modeling was written in Python3 (version 3.9.5) using the pandas, numpy, matplotlib, seaborn, and scipy libraries (Hunter 2007, McKinney 2010, Harris *et al.* 2020, Virtanen *et al.* 2020, Waskom 2021).

### 2.1 Processing of 3did content

3did content was downloaded from3did.irbbarcelona.org/download/current/3did_flat.gz. The downloaded file contains DDI interfaces identified in 3D structures of the PDB. Multiple interfaces can be related to the same DDI type, if the same two domains are found to be in contact with each other. Each interface is uniquely identified by the Pfam–Pfam combination of the interacting domains, the PDB structure it was detected in as well as the ID(s) of the chain(s) the domains were detected in. Furthermore, 3did provides their 3did score and *z*-score for each interface, as well as the interacting residues for both domains involved in the DDI type. The number of residue–residue contacts per DDI type was derived from the list of contacting residues of the domains mediating the interaction for the interface with the highest 3did score. Intrachain DDI types were defined as being supported only by structures exhibiting intrachain interfaces. If both domains mediating the interaction had the same chain ID, the interface was classified as intrachain. Homo-protein DDI types were defined to be only supported by homodimeric interfaces between the same protein UniProt ID. To this end, we retrieved the UniProt IDs of the proteins present in structures with interfaces formed by a certain DDI type along with the respective chain IDs using the GraphQL-based API of the RCSB PDB. If multiple interfaces of the same DDI type are present in one structure, we selected the interchain interface with the highest 3did score of this structure. If no interchain interface was present in a structure, the intrachain interface with the highest 3did score was kept as the representative interface for the structure.

### 2.2 Development of a manual curation standard and manual curation

Please refer to Supplementary Material.

### 2.3 Extraction of CATH annotations for domains in benchmark dataset

For the mapping of CATH to Pfam domains forming the DDIs in the manual curation set, we extracted the 3D structure with the highest 3did score for each DDI type, along with the residues involved in residue–residue contacts between both Pfam domains. Matching CATH domains in the proteins of the respective PDB structures including the start and end of each match were obtained from the CATH database (Orengo *et al.* 1997, Sillitoe *et al.* 2021). Afterwards, overlaps between CATH and Pfam domains were identified by contacting residues overlapping within the CATH domain matches.

### 2.4 Feature annotation for machine learning

Please refer to Supplementary Material.

### 2.5 Logistic regression

Model training and analysis was performed with code written in R version 4.3.0. The logistic regression and leave-one-out cross validation were performed using R base functions. Odds ratios are obtained from the logistic model estimates through the function *f*(*x*) = *ex*, where *e* = 2.72, and *x* is the estimate from the logistic model (Agresti 2012). ROC curves and optimal thresholds for calculating sensitivity and specificity (determined using Youden’s criterion) were computed using the package pROC version 1.18.2. True positives are defined as DDI types from the benchmark dataset that we approved and were predicted as such. True negatives are curated DDI types that were not approved and also did not pass the cut-off from logistic regression. False positives and false negatives are non-approved and approved DDI types from the benchmark dataset that passed or did not pass the cut-off from the model, respectively. The package glmtoolbox version 0.1.7 was used to compute Hosmer-Lemeshow goodness-of-fit tests. All codes are available at https://github.com/KatjaLuckLab/DDI_manuscript including the feature table for model training.

### 2.6 GO enrichment analysis

We extracted the Ensembl gene IDs of all proteins in the HuRI dataset that were subsequently mapped to Entrez GeneIDs using the mygene package (Wu *et al.* 2013). The GO term enrichment analysis for the subset of proteins in HuRI forming interactions for which a DDI was predicted, was performed in R version 4.3.2 using the clusterProfiler (Yu *et al.* 2012, Wu *et al.* 2021) and DOSE (Yu *et al.* 2015) libraries while using the whole HuRI dataset as background.

### 2.7 Structural modeling of predicted DDIs using AlphaFold and integration with Burke *et al.* and Interactome3D data

Computed pDockQ values for PPIs modeled in Burke *et al.* (2023) were downloaded from https://archive.bioinfo.se/huintaf2/. To merge data between this downloaded dataset and the predictions obtained in this study, the Ensembl IDs for each chain within the Burke dataset were mapped to UniProt/SWISS-PROT IDs using the UniProt ID mapping feature (The UniProt Consortium 2023). Interactions with the same pair of UniProt IDs were then paired and model characteristics were merged based on this pairing. The fraction of disordered residues in each protein sequence was computed as described below using IUPred. Annotations of human PPIs with structural information downloaded from Interactome3D on 21 March 2024 (Mosca *et al.* 2013). PPIs with annotation “Structure” were considered as PPIs for which a structure has been resolved. The structural modeling of predicted DDIs was performed using AF-Multimer v2.3 on protein fragments corresponding to the predicted DDIs. While 3did provides domain boundary annotations from Pfam HMM matches, these domain boundary annotations sometimes fail to cover the whole fold of the domains. This represents a problem for accurate modeling by AF. To circumvent this, a curated list (Lee *et al.* 2024) of one representative domain structure from each of 136 domain–motif interface classes was used to derive a pLDDT cut-off that defines order-to-disorder boundary transitions. Briefly, the domain structures from the domain–motif interface classes were superimposed onto the AF-predicted full-length monomeric structures of the domain proteins. Then, the pLDDTs of the 10 residues within and outside of the domains were averaged. By assessing the distributions of these averaged pLDDTs, we derived a pLDDT cut-off of 80 that corresponds to order-to-disorder boundary transitions. To use this pLDDT cut-off to improve the domain boundary annotations from Pfam matches, we calculated the average of the pLDDT of 10 residues within Pfam matches and checked if the averaged pLDDT was lower than 80. Should the averaged pLDDT be higher than 80, we would extend the domain boundaries by one residue and re-calculate the average pLDDT. This process was performed iteratively until either the cutoff was reached or the boundaries reached the protein termini. AF was run as described above.

### 2.8 Assessment of disorder propensity at interfaces in Burke *et al.* and reference datasets

Three datasets were analyzed: a curated list (Lee *et al.* 2024) of one representative structure from each of 136 domain–motif interface classes, AF-Multimer structures (Burke *et al.* 2023) with pDockQ > 0.5, and the representative structures of the 48 approved DDI types. For each AF-Multimer structure, the list of interacting residues was parsed from the pLDDT files accompanying the structure files within the data download from https://archive.bioinfo.se/huintaf2/. For the other two datasets, interacting residues were identified by parsing the structure files with the Biopython PDB module (Cock *et al.* 2009) and calculating interatomic distances. Those residues with an atom within 10 Å of the other chain were designated as interacting, in accordance with the definition used for AF-Multimer models (Burke *et al.* 2023). Residue numberings were shifted from PDB structure indexing to UniProt sequence indices using the SIFTS PDB-UniProt residue-level mapping database (Velankar *et al.* 2013, Dana *et al.* 2019). Residue-level disorder propensity was obtained by submitting the Uniprot accession ID for each protein chain to the IUPred2A API (Mészáros *et al.* 2018). For structures from the Burke dataset, the annotation of UniProt IDs is described above. The fraction of predicted disorder within a chain was calculated by dividing the number of interacting residues in the chain with IUPred2A > 0.4 by the total number of interacting residues within the same chain. Whole interface disorder propensity was calculated similarly but with the total set of interacting residues within an interface.

## 3 Results

### 3.1 Generation of a reference dataset of DDI types

To generate a manually curated reference set of DDI types, we aimed to randomly select a subset of DDI types from 3did and subject the structures and corresponding publications to manual inspection. In 3did, residue contacts are computed between Pfam HMM matches occurring in the same or different protein chains. 2220 DDI types in 3did (15%) have only intrachain evidence. While there is evidence that some domain types found to be in contact with each other within a protein chain can mediate a protein interaction when situated in distinct protein chains (see also Section 4), it is impossible to evaluate intrachain DDI types for this ability using available intrachain structures and corresponding publications. Given limited manpower and time, we decided not to extend our manual curation to experimental evidence beyond the solved structures and therefore opted to restrict the generation of a reference dataset of DDI types to those with interchain evidence as reported in 3did.

**Figure 1.**
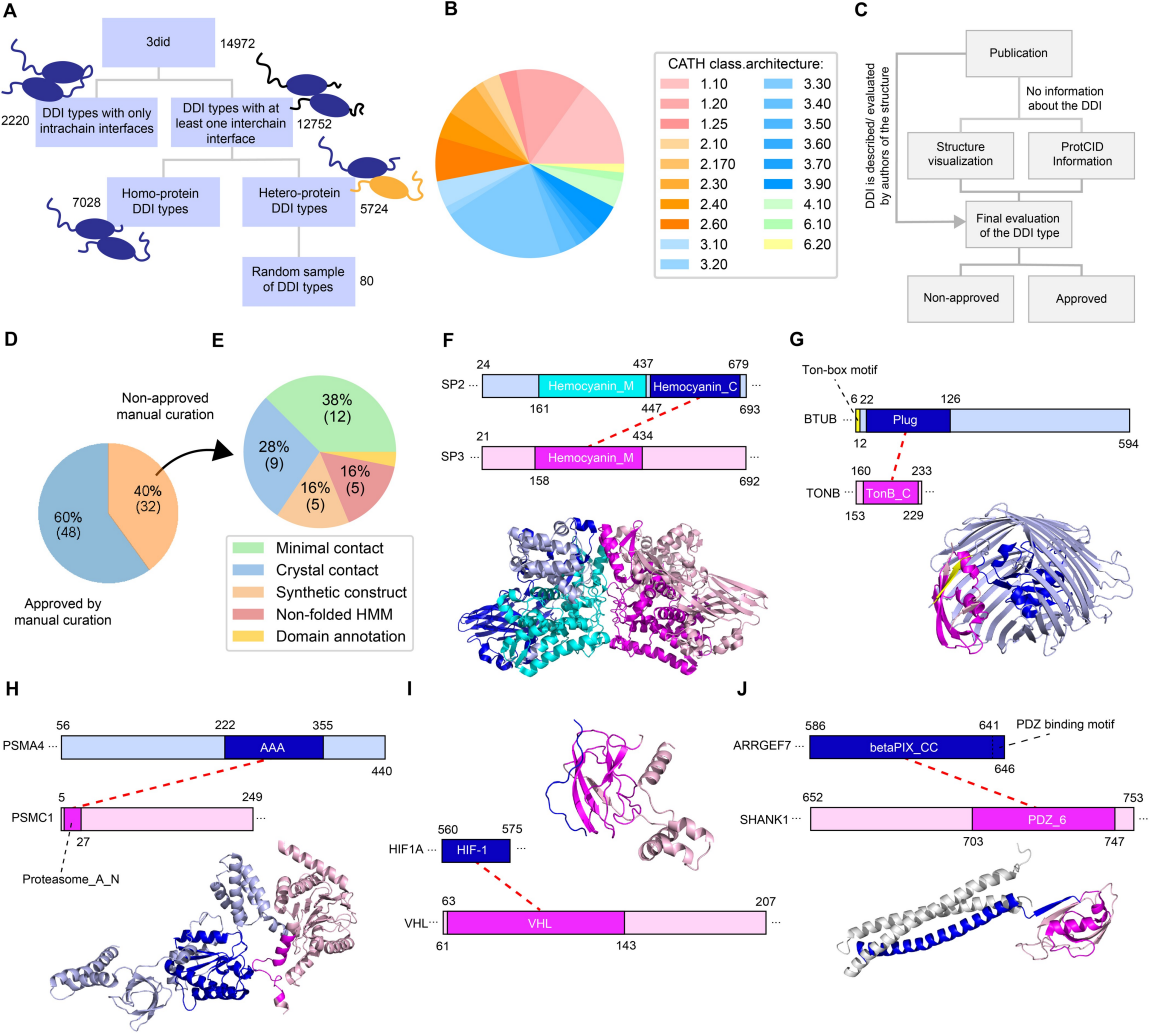
Classification of DDI types in the 3did database and manual curation. (A) Schematic describing the classification of DDI types from 3did and from which subset the sample of DDI types were drawn for manual curation. (B) Representation of CATH classes and architectures among domain folds in the randomly sampled DDI types. (C) Schematic describing the manual curation procedure. (D) Pie chart showing the fraction of approved and nonapproved DDI types from manual curation. (E) Pie chart showing categories and fractions of nonapproved DDI types. (F–J) Exemplary structures and domain architectures of protein pairs from nonapproved DDI types. Thick dashed lines mark the DDI as reported in 3did. Structures were obtained from PDB IDs 3wjm, 2gsk, 6epf, 6gfx, and 3l4f.

One of the challenges in crystallography, the method that was used to determine the vast majority of protein structures deposited in the PDB, is the discrimination of biological contacts between protein molecules from those that are solely driven by the crystallization process. While the so-called crystal contacts can hint at interfaces between proteins occurring under high concentration conditions, for the generation of a high-confidence set of DDI types, we opted to exclude DDI types that are likely based on crystal contacts alone. Discrimination between biological assemblies and crystal contacts is particularly difficult for contacts observed between molecules of the same protein, i.e. homodimers, a task with which even structural biology experts struggle (Elez *et al.* 2020, Schweke *et al.* 2023). We found that 60% (7028) of the DDI types in 3did with at least one interchain interface in a PDB structure are only derived from structures of homodimers, which we felt unconfident to evaluate. This leaves us with 5724 DDI types with interchain evidence and at least one structure with a heterodimeric interface, which we considered further for the generation of a reference dataset of DDI types (Fig. 1A).

We randomly selected 80 heteroprotein DDI types with interchain evidence for manual inspection. To assess the structural diversity of the selected domain types, we assigned folds to the CATH hierarchy and found that all five classes as well as 19 architectures are well represented among the selected DDI types (Fig. 1B). Nine domain types participated in more than one DDI (Supplementary Fig. S1A) following domain-type distributions across the whole subset of 5724 DDI types (Supplementary Fig. S1B). We approved a DDI type after manual inspection if there was at least one structure supporting that the given PPI was mediated by contacts between both domains. In order to evaluate a DDI type, we assessed the publication (if available) reporting a structure for evidence that the two domains are mediating the PPI. We also inspected the residues observed to be in contact with each other and their overlap with the corresponding Pfam HMM matches. We further evaluated to which extent the HMM matches corresponded to folded domains and if most contacts were mediated by residues belonging to these folded regions. Lastly, we also evaluated to which extent the reported interface was observed in other structures as reported in the ProtCID database (Fig. 1C, see also Section 2).

3did computes a score and its significance as a *z*-score for every DDI type. These scores reflect the significance of the interface in terms of type and number of contacts observed between residues of both Pfam HMM matches in comparison to random observations. The higher the score and *z*-score, the more reliable the DDI type. For each of the 80 selected DDI types, we ranked all structures from the highest to the lowest 3did score and inspected the top-ranked structures. We quickly realized that either the top-ranked structure was approved or none of the structures were. We therefore evaluated at most the top two ranked structures for every DDI type. In total, 95 structures were manually inspected with an average of 2 h spent per structure. Most structures (67%) were determined by X-ray crystallography with a resolution spanning 1.5–11.8 Å (mean 3 Å, Supplementary Fig. S1C). This work resulted in the approval of 48 of 80 potential DDI types (60%) (Fig. 1D). Of note, non-approval of a DDI type does not necessarily mean that this DDI type cannot mediate a PPI, but the available structural data currently does not provide enough evidence. Detailed reasoning for approval and non-approval is provided for every DDI type and inspected structure in Table S1.

Often a DDI type was not approved because the observed domain-domain contacts were considered too few to mediate the PPI (12 DDI types, on average 7.6 contacts, Fig. 1E). In these cases, we observed contacts between other domains or neighboring linear motifs in the same structure that were much more likely to mediate the PPI. For example, a structure between the two Bombyx mori proteins SP2 and SP3 shows a strong interaction between the Hemocyanin_M domains of both proteins, which is also discussed in detail in the accompanying study (Hou *et al.* 2014). The contact between the Hemocyanin_M domain in SP3 and the Hemocyanin_C domain in SP2 is, however, very small (11 contacts, Fig. 1F). This interface is not further discussed in the corresponding publication but is reported as a DDI type in 3did. In another case, 3did reports a DDI type between the Plug domain of BtuB and the TonB_C domain of TonB from *Escherichia coli*. The contact between both domains is minimal and involves part of a disordered region, which is unlikely to belong to the fold of the Plug domain. The main contact, which is well described in the literature, is established between a linear motif called the Ton-box located N-terminal to the Plug domain, binding in beta-strand augmentation to TonB_C (Fig. 1G) (Shultis *et al.* 2006). For nine other DDI types, the observed domain–domain contacts were likely a result from crystallization only (Fig. 1E). For example, for DDI types such as PF00019_PF00041 and PF12139_PF02910, the authors of the respective publications studied the corresponding interfaces and dismissed their biological relevance (Fritz *et al.* 2002, Healey *et al.* 2015).

For five additional DDI types, reported contacts by 3did between protein fragments indeed likely mediate the interaction observed in the structure; however, at least one of the two interacting protein fragments does not correspond to a tertiary folded domain. For example, the Pfam HMM Proteasome_A_N (PF10584) matches in the N-terminal disordered region of PSMA4, which mediates binding to the AAA domain (PF00004) in protein PSMC1 (Fig. 1H). In another case, the Pfam HMM corresponds to the known prolyl hydroxylation short linear motif in hypoxia-inducible factors (HIFs) (Fig. 1I), which is recognized by VHL leading to the ubiquitination and degradation of HIF (see also the entry DEG_ODPH_VHL_1 in the ELM DB). The motif sits in a disordered region of HIFs. Of note, the Proteasome_A_N and HIF-1 HMMs are classified as “family” and “domain” HMM types by Pfam, respectively. We also found a case where the Pfam HMM betaPIX_CC (PF16523) comprises a coiled–coil region and a C-terminal PDZ-binding motif. The DDI reported by 3did is clearly mediated by the linear motif in the protein ARHGEF7 binding in beta-strand augmentation to the PDZ domain of SHANK1 (Fig. 1J). For five of the inspected DDI types we found that existing folds were further engineered to bind new targets. Ankyrin repeat domains are commonly used to develop DARPINs while V-set domains are used in antibodies and nanobodies to bind antigens. DDI types PF00023_PF07686 and PF00571_PF07686 are two examples of such synthetic interfaces, which should not be used to predict DDIs in physiological PPIs. Among the 32 nonapproved DDI types was also one instance (PF11831_PF08231) where reported Pfam HMM and PDB chain annotations in 3did were wrong.

### 3.2 Feature annotation for machine learning

**Figure 2.**
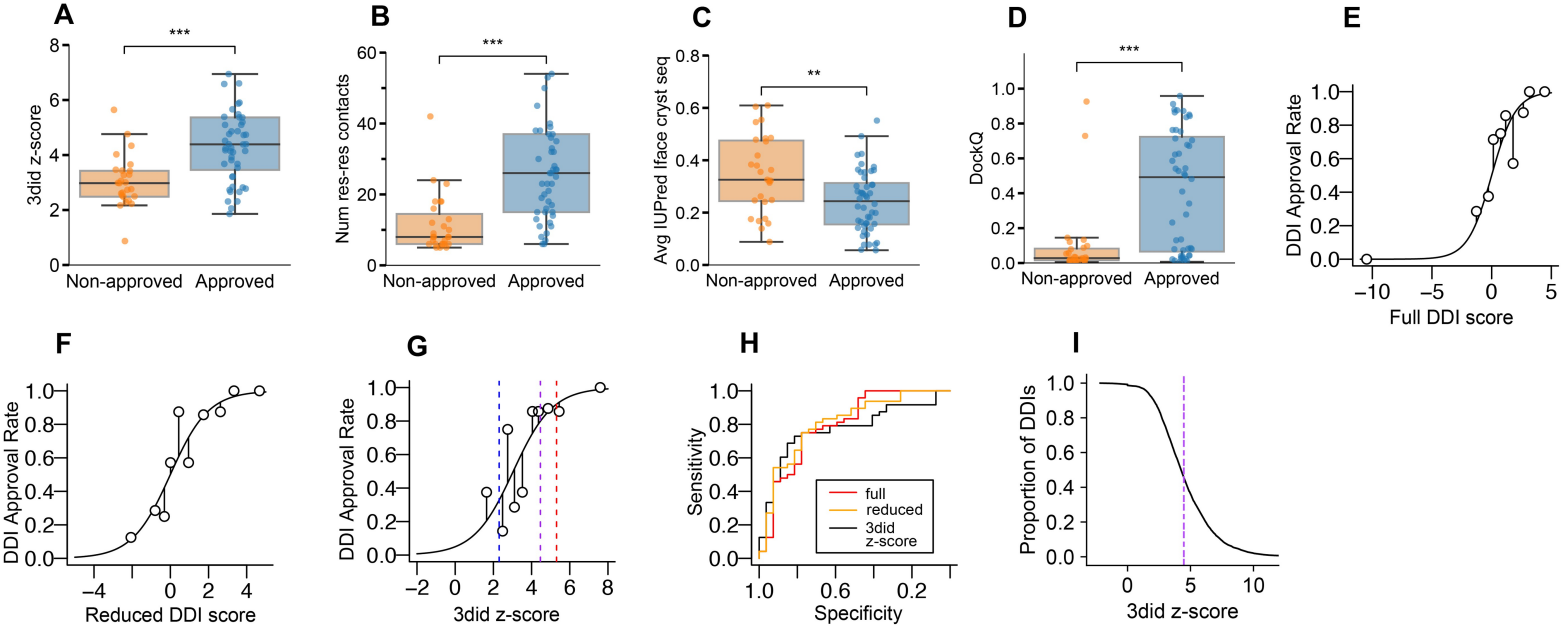
Model training and prediction of highly confident DDI types. (A–D) Boxplots showing the distribution of approved and nonapproved DDI types for different features as indicated on the *y*-axis. Significances were computed using the Mann–Whitney *U* test. (E–G) Empirical fit assessment is shown for the full (E), reduced (F), and minimal model (G). The DDI training dataset was split in 10 groups by deciles and for each group the proportion of approved DDI types is shown. The *z*-score threshold of 2.3 (*y* = 0.31), 4.47 (*y* = 0.8), and 5.30 (*y* = 0.9) are plotted in panel G. (H) ROC curves for all three models computed using leave-one-out cross-validation. (I) Proportion of heteroprotein DDI types with interchain evidence in 3did retained based on increasing 3did *z*-score cut-offs. The vertical line indicates the suggested 4.47 3did z-score cut-off.

Given the considerable number of nonapproved DDI types in our curation dataset, the time needed to manually check a DDI type, and the considerable number of heterodimer-derived DDI types with interchain structures in 3did, a clear need arises to computationally identify high confident DDI types. To train a classifier for this task, we set out to determine features for DDI types that would serve as input for the classifier. We extracted interface-related features from 3did and ProtCID, computed disorder propensity of interface residues using IUPred, explored enrichment for domain pairs in interacting proteins, and modeled structures of interacting domain pairs using AF (Supplementary Dataset S1). We found that features reflecting the number of contacts between interacting domain pairs, i.e. the 3did *z*-score, distinguished well between approved and nonapproved DDI types, as expected (Fig. 2A–B). Nonapproved DDI types also tended to display a higher disorder propensity for interface residues (Fig. 2C). Interestingly, AF could much more readily model structures of interacting domain pairs, as assessed by higher DockQ scores when compared to resolved structures, if those belonged to approved DDI types (Fig. 2D). However, features extracted from ProtCID had no significant discriminative power nor did features extracted from PPI networks (Supplementary Fig. S2 and Section 2). In our curation effort, the most important feature supporting a DDI type was its description in an accompanying publication of the structure. We employed text mining to identify the cooccurrence of domain names in abstracts but realized that available domain names from the Pfam database are rarely similar (and essentially never identical) to domain names used by experimentalists studying these domains (Supplementary Table S2). We therefore had to exclude text mining of abstracts as a feature to predict approval of DDI types. Of note, for the aforementioned feature analyses as well as for the downstream modeling we removed all DDI types from the benchmark dataset (5 in total) that involved an Ankyrin or V-set domain since they are commonly used in protein design and thus most often, will not correspond to naturally occurring DDIs. The benchmark dataset with all feature annotations is available in Supplementary Table S3.

Given the small size of our DDI benchmark dataset in comparison to the large number of hetero-protein DDI types in 3did, we assessed to which extent the mean of the annotated features differed between both sets of DDI types and found that all features apart from the 3did score, 3did *z*-score, and number of residue–residue contacts did not significantly deviate in their mean (Supplementary Fig. S3).

### 3.3 Feature selection, training, and testing of logistic regression model

We analyzed the correlations among the features to sort out redundant variables (Supplementary Fig. S4A–B). Given the small training dataset, we opted to use logistic regression to predict DDI approval. We refer to this model as the full model, because it includes the largest set of variables (Supplementary Table S4 and Supplementary Fig. S4B). To simplify the model we used backwards stepwise feature selection to keep only the most informative variables. We also excluded the number of intrachain structures from the model as its regression coefficient had a very large standard error, its p-value was very close to 1, and it caused convergence issues (Supplementary Table S4). The resulting reduced model consisted of the 3did *z*-score, AF DockQ, and the IUPred score (Supplementary Table S4, Fig. S4C). We also tested a minimal model consisting only of the 3did *z*-score to assess if addition of any of the features improved DDI approval prediction compared to using the 3did *z*-score alone (Supplementary Table S4). The full model fits significantly better than the minimal model (*P* = .007; chi-square test), whereas the reduced model was not significantly inferior to the full one (*P* = .336; chi-square test, Fig. 2E–G). We assessed the predictive power of the models through ROC curve analysis using leave-one-out cross-validation (Fig. 2H). A bootstrap test failed to identify significant differences in performance among the models (minimal vs. full: *P* = .58; minimal vs. reduced: *P* = .32; full vs. reduced: *P* = 0.61). The area under the curve was 0.795, 0.807, and 0.772 for the full, reduced, and minimal model, respectively. This suggests that, although the reduced model fits the training data better than the minimal model, with the current benchmark dataset we could not demonstrate a significant improvement in prediction accuracy by the addition of further variables. The overall predictive power of all three models when considering the optimal threshold was found to be in the range of 78–81% sensitivity and 73–75% specificity.

The 3did *z*-score is a robust predictive feature in our analysis. The minimal model indicates that every increase of the 3did *z*-score by one unit roughly doubles the odds of approval for a DDI type (Supplementary Table S4). The authors of 3did suggest using a 3did *z*-score of 2.3, which, however, is not applied by default to 3did content. We found that a *z*-score cut-off of 2.3 results in an expected DDI type approval probability of 33%. Only much higher *z*-score cutoffs of 4.47 and 5.30 can reach a desired approval probability of 80% and 90%, respectively. Applying a 3did *z*-score cut-off of 2.3, 4.47, or 5.3 retains 87%, 46%, or 32% of the hetero-protein DDI types with interchain evidence in 3did (Fig. 2I and Supplementary Fig. S4D–E and Table S5).

### 3.4 Prediction and structural modeling of DDIs in the human protein interactome

**Figure 3.**
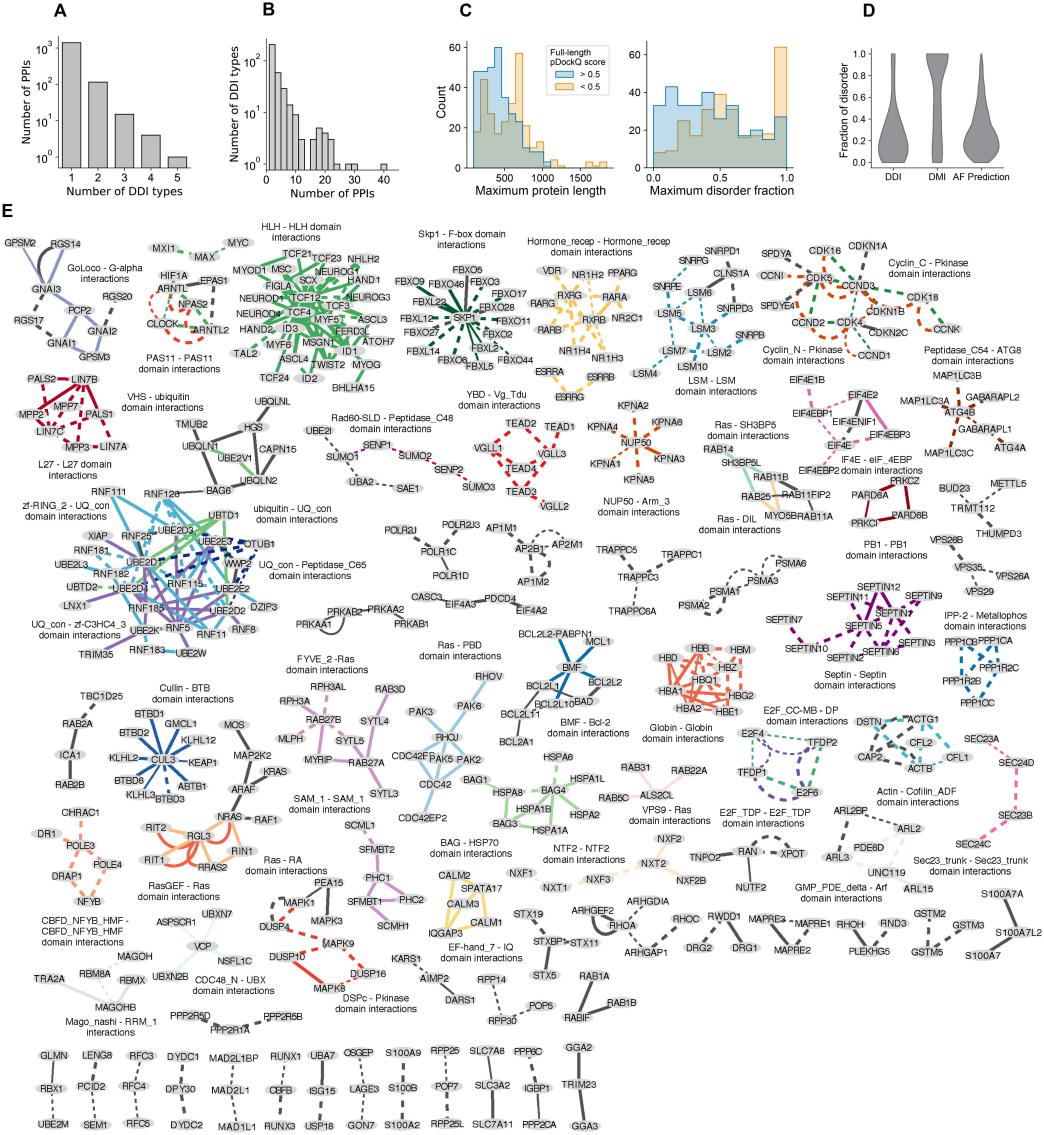
DDI prediction in HuRI and structural modeling. (A) Histogram showing how many PPIs had how many different DDI types predicted to mediate a given PPI (outliers excluded from the plot). (B) Histogram showing how many DDI types were predicted to occur in how many PPIs. (C) PPIs for which a highly confident structural model was obtained using predicted interacting domain fragments were split based on their pDockQ score in Burke *et al.* (2023) and plotted as a histogram based on the maximum protein length and maximum fraction of disordered residues in the full-length chains of both partner proteins. Differences in distributions are significant (Mann–Whitney *U* test: *U* = 31 308, *P* < .0001 for length; *U* = 30 194, *P* < .0001 for disorder fraction). (D) Violin plots are shown for the maximum fraction of disordered residues among interface residues for both partner proteins based on resolved structures from the DDI benchmark dataset from this study, a DMI benchmark dataset (Lee *et al.* 2024), and AF-derived structural models from Burke *et al.* (2023) at a pDockQ cut-off of 0.5. (E) Network of PPIs from HuRI with predicted and successfully structurally modeled DDIs. Shown are PPIs with the most commonly predicted DDI types. Edges are colored by DDI types, which are also labeled. Nodes are labeled with gene symbols. Thin and thick edges correspond to those for which a resolved or no structure is available, respectively. Dashed and solid lines indicate PPIs for which a high confident AF model is or is not available from using full-length protein sequences as reported in Burke *et al.* (2023).

To explore the usefulness for guiding AF complex structure modeling using predictions of likely interacting domain fragments, we referred to HuRI, a systematically generated human protein interactome dataset obtained from yeast two-hybrid screens (Luck *et al.* 2020). We scanned protein sequences of interacting proteins in HuRI for HMM matches corresponding to high-confident DDI types. We found that more stringent 3did *z*-score cutoffs resulted in larger differences between the number of PPIs with a predicted DDI in HuRI versus degree-controlled randomized networks (Supplementary Fig. S5). This suggests that higher 3did *z*-score cutoffs enrich for functional DDI types. Using a 3did *z*-score cut-off of 4.47, we predicted at least one DDI for 1533 PPIs in HuRI (Supplementary Table S6). These PPIs show no particularly pronounced enrichment for gene ontology terms (Supplementary Fig. S6). For 135 of these PPIs, more than one possible DDI was predicted (Fig. 3A). In total, 342 DDI types resulted in at least one prediction in HuRI and on average a DDI type was predicted to mediate about 2–5 PPIs (Fig 3B). We realized that for 381 of these PPIs, the predicted DDI type corresponds to coiled-coils formed by rod domains (PF00038) occurring mostly in intermediate filament proteins such as keratins. To avoid substantial bias in downstream analyses, we decided to remove these PPIs and DDI predictions for subsequent modeling by AF. For the remaining PPIs we subjected the predicted interacting domain fragments for complex structure modeling to AF and were able to obtain a structural model for 1129 PPIs (Supplementary Table S6). To identify highly confident structural models, we applied a model confidence cutoff of 0.8 for which it was previously estimated that the false and true positive rate equaled zero and 31%, respectively (Lee *et al.* 2024). At this cut-off, we obtained a highly confident structural model for 604 PPIs (Supplementary Dataset S2); 394 of which, according to Interactome3D, have no available resolved structure (Supplementary Table S6). To assess to which extent structural modeling by AF was aided by the use of predicted interacting domain fragments versus the use of full-length proteins, we referred to AF-derived structural models published in Burke *et al.* (2023). Using full-length sequences, Burke *et al.* reported a highly confident structural model (pDockQ > 0.5) for 320 out of the 604 PPIs, meaning that for almost half of all PPIs, guiding AF by pointing to likely interacting protein fragments was essential to obtain a highly confident structural model. PPIs for which highly confident structural models were only obtained using protein fragments tended to consist of proteins that are significantly longer and more disordered (Mann–Whitney *U* test: *U* = 31 308, *P* < 0.0001 for length; *U* = 30 194, *P* < .0001 for disorder fraction) (Fig. 3C, Supplementary Table S6). Interestingly, the fraction of well-modeled PPIs with a DDI using full length sequences was still much higher compared to a 5% success rate observed for interfaces mediated by short linear motifs binding to folded domains (Lee *et al.* 2024). This is in contrast to earlier findings reporting that domain–motif interfaces (DMIs) and DDIs could be modeled equally well by AF when using protein fragments (Lee *et al.* 2024). Indeed, assessment of the confidently modeled interfaces reported in Burke *et al.* (2023) using full length sequences showed a strong tendency towards ordered interfaces (Fig. 3D) suggesting that interfaces involving disordered regions are particularly missed when using full length sequences for structural modeling by AF.

**Figure 4.**
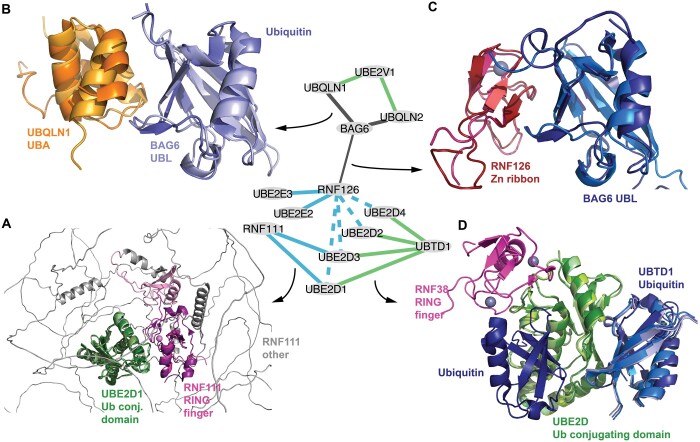
Selected predicted and modeled DDIs relating to protein quality control functions. (Middle) Subnetwork from Fig. 3E shown, color-coding as in Fig. 3E. Arrows indicate PPIs shown with structural models in individual subpanels. (A) Superimposition of one resolved structure and two AF models: (i) structure of RNF12 RING finger domain binding to UBE2E2 Ub conjugating domain (6W9A, dark colors), (ii) AF model of RNF111 RING finger domain binding to UBE2D1 Ub conj. domain (middle colors), (iii) AF model retrieved from Burke *et al.* (2023) with full-length sequences of RNF111 and UBE2D1 (light colors). In the latter model, the RING finger domain (light pink) is not well aligned with the other AF model and structure (middle and dark pink). (B) Superimposition of a resolved structure (2JY6, dark colors) showing UBQLN1 UBA domain binding to mono-ubiquitin with an AF model of UBQLN1 UBA domain binding to BAG6 UBL domain (light colors). (C) Superimposition of a resolved structure (2N9P, dark colors) showing the RNF126 Zn ribbon domain binding to the BAG6 UBL domain with an AF model of the same two proteins and domains (lighter colors). Of note, the BAG6 UBL domains in B and C are displayed in the same orientation based on superimposition. (D) Superimposition of one resolved structure with four AF models: (i) structure (4V3L) of RNF38 RING finger domain (pink) binding to UBE2D2 Ub conj. domain (dark green), which itself is bound by two mono-ubiquitins (dark blue), one close to the catalytic side (left) and one on the back of the Ub conj. domain (right), (ii–iv) AF models of UBTD1 Ubiquitin domain (shades of blue, left) binding to Ub conj. domains of UBE2D1/2/3/4 (shades of green).

PPIs with predicted and confidently modeled structures of DDIs involving 206 different types are shown in Fig. 3E and Supplementary Fig. S7. Focusing on the most commonly recurring DDI types, we observe associations with regulatory and signaling functions involving ubiquitin-like, RING fingers, BTB, Fbox, Skp1, and Cullin domain interactions relating to protein quality control, helix-loop-helix, nuclear, and hormone receptor domain interactions relating to gene expression, and DDIs involving the Ras domain relating to G-protein signaling. Focusing on DDI predictions in protein quality control, we obtained several novel structural models for interactions between E3 RING ligases and E2 enzymes involving predicted RING finger domain—Ubiquitin-conjugating domain interactions (zf-RING—Ub-con, PF13639-PF00179). Superimposition between the structural model obtained with domain fragments and resolved structures of zf-RING—Ub-con complexes shows high structural similarity of the interfaces in contrast to structural models obtained using full-length sequences (Fig. 4A). We furthermore predicted a DDI involving the BAG6 Ubiquitin-like (UBL) domain and the UBQLN1 Ubiquitin-associated (UBA) domain akin to structurally resolved UBA-Ubiquitin interactions (Fig. 4B). Interestingly, the structurally resolved interface between the BAG6 UBL domain and the RNF126 zinc-ribbon domain (Krysztofinska *et al.* 2016) (which was accurately modeled by AF using protein fragments, Fig. 4C) overlaps substantially with the UBL-UBA interface suggesting that BAG6 interactions with RNF126 and UBQLNs are mutually exclusive (Fig. 4B and C). This hypothesis might further help elucidate potential modes of crosstalk between UBQLNs and BAG6-mediated quality control of mislocalized proteins (Kong *et al.* 2021). Finally, we predicted DDIs between the Ubiquitin (Ub) domain of UBTD1 and the Ub-con domain of three UBE2D-type E2 enzymes (Fig. 4D). A previous study has shown that UBE2D2’s catalytic activity is enhanced upon noncovalent binding of a Ub molecule to the Ub-con domain opposite to where another Ub molecule is conjugated onto Ub-con prior to transfer onto protein substrates (Buetow *et al.* 2015). Interestingly, AF predicted the Ub domain of UBTD1 to bind to the allosteric activation site of the Ub-con domain (Fig. 4D) providing a mechanistic hypothesis for previously reported roles of UBTD1 to enhance the degradation of MDM2 and YAP (Zhang *et al.* 2015, Yang *et al.* 2020).

## 4 Discussion

Striving for structurally resolved protein interactomes is a goal that has become much more attainable in the era of AI-driven protein structure prediction. As outlined by findings reported previously and in this study, there are multiple obstacles on the path to this goal; however, there are also potential avenues to resolve them (Burke *et al.* 2023, Lee *et al.* 2024). Here, we showed that the sensitivity of AF protein complex modeling can be increased when guiding AF with information on likely interacting protein fragments. A prerequisite for this approach to work is access to high confidence lists of interface types. We established a reference set of manually curated DDI types and used it to train a logistic regression model that enabled prediction of likely high confidence DDI types in 3did. Of note, we based our curation on DDI types with interchain evidence and contacts observed between heterodimers. We had excluded all DDI types from 3did that were derived from structurally resolved homodimers due to particular difficulties in distinguishing biological assemblies from crystal contacts for this class of PPIs. In the future, specific models trained for this task could be incorporated to curate and rank this class of DDIs (Schweke *et al.* 2023). We also systematically excluded DDI types that only have structural evidence from intrachain contacts. Some of these excluded types could be added by considering experimental studies beyond the solved structures. For example, we are aware of many intramolecular modes of binding that serve functionally important autoinhibitory roles. There is also evidence from studying mainly unicellular organisms that interacting domains from distinct proteins in some species evolved via protein fusion to become part of a single protein chain in other species. Furthermore, artificial protein fusions are sometimes used in crystallography to enhance crystallization. By extending the manual curation process to these types of evidence, a fraction of intrachain DDI types could be added to the set of high confidence DDI types in the future.

We acknowledge that our DDI benchmark dataset is small due to limited time and manpower available to curate DDI types. It is possible that because of the limited size of the training dataset, significant differences between the trained models could not be observed. A larger training dataset might have enabled application of more sophisticated machine learning models resulting in higher prediction accuracies. Despite these limitations, our curation effort and subsequent analyses have provided important insights and avenues to improved DDI type detection as outlined below. We found that nonapproval of DDI types often resulted from Pfam HMM matches not corresponding to folded domains as well as inaccurate prediction of domain boundaries. Pfam HMMs are classified into family, domain, repeat, and signal types, all of which are considered in the search for domain–domain contacts in 3did to the best of our understanding. However, the Pfam HMMs that we found matching to disordered regions are classified as family or domain type, meaning that a restriction to these HMM types would not have helped identifying these cases. Accurate prediction of domain occurrences and boundaries likely requires a structure-based approach as implemented by CATH. CATH provides a classification of protein folds that they recently expanded thanks to releases of AF structural models across hundreds of proteomes. Using clusters of highly similar folds, they built sequence search models that were found to accurately predict domain occurrences and boundaries in protein sequences (Sillitoe *et al.* 2021, Bordin *et al.* 2023, van Kempen 2023). Along with these proposed improvements for domain discovery, our work also highlighted the importance of selecting biologically relevant interfaces in resolved protein structures for DDI type inference. Our work suggests that the number of residue–residue contacts observed between both chains as well as abilities of AI tools like AF to accurately model these interfaces seem to be relevant features to achieve this task, in line with findings reported elsewhere (Schweke *et al.* 2023). Identification of biologically relevant interfaces might also be aided by considering interface features computed by PDBePISA (Krissinel and Henrick 2007).

We noted that the use of text mining to automatically extract DDI information from publications failed because there is essentially no consensus on the naming of folded domains. While a widely applied standard for the naming of types of functional modules in proteins would be highly beneficial in many respects, we also would like to argue that DDI type inference should ultimately be structure- and not text-based. In this sense, we see our approach to define a high confidence set of DDI types as an intermediate solution and laid out clear avenues for improved DDI type inference in future studies.

Guiding AF protein complex structure predictions with sequence pattern-based DDI predictions increased the number of PPIs with a high confidence AF model from 40% to 65%. In addition, this approach readily provides functional annotations for the predicted interface that are derived from the DDI type and corresponding resolved structures. Focusing on predicted interfaces between proteins functioning in protein quality control, we generated intriguing mechanistic hypotheses that can guide future experimental work, highlighting the power of this approach. However, guiding AF predictions with information on known interface types of course will only be applicable to PPIs mediated by instances of those. Assessment of interface properties of highly confident AF structural models obtained using full-length protein sequences revealed substantial biases towards DDIs, highlighting the importance for considering different modes of protein binding when assessing structural modeling capabilities. In summary, this study highlights the need and potential of integrative structural biology approaches to get us closer to structurally resolved protein interactomes.

## Supplementary Material

btae482_Supplementary_Data
