# Supplementary material for: Generation of a high confidence set of domain–domain interface types to guide protein complex structure predictions by AlphaFold
Source: Bioinformatics. 2024 Aug 22;40(8):btae482. doi: 10.1093/bioinformatics/btae482 (PMC11361816; doi:10.1093/bioinformatics/btae482)
Supplement: btae482_Supplementary_Data [file btae482_supplementary_data.zip › Supplementary_information.docx]

**Supplementary Methods**

### *Development of a manual curation standard and manual curation*

For the manual curation of the 80 randomly selected DDI types, we primarily relied on assessing the publication(s) of the structures exhibiting a DDI interface (if they were available). This was conducted by searching for a description of the DDI, either affirming the interaction between the respective regions or identifying the contact as a crystallographic artifact. We did not require the authors to refer to the specific Pfam domains but accepted the description of an interaction between the regions corresponding to the Pfam domains. Furthermore, we evaluated information provided in the publication about interaction stoichiometries, the localization of both proteins, and other functional background of the interaction. This information was especially helpful to evaluate the DDI type, if the DDI itself was not described in the publication. We also investigated whether the interacting proteins were the result of synthetically engineered protein scaffolds or whether the structure contained a specifically designed antibody binding its target protein. If the DDI was described as a functional interface meditating the PPI within the publication, we considered this as sufficient to evaluate the DDI type as approved. Following the assessment of the publication(s), we continued the curation process by inspecting the residue-residue contacts between the domains of the DDI using the PyMOL Molecular Graphics System (version 2.5.0). The ProtCID database provides metrics for interaction interfaces to aid in the identification of protein contacts that likely originated from crystallization. ProtCID is based on the assumption that conservation of biological assemblies among homologous proteins identified in different crystal forms is unlikely to be the result of crystallization alone. They provide clusters of interfaces present in PDB structures in at least two different crystal forms, with further annotations including the PDB structures contributing to the cluster, the UniProt IDs of the proteins in the structures, the minimal sequence identity of the proteins in the cluster or the crystal forms of the PDB structures in the cluster. As the final step in our manual curation, we queried the web server of the ProtCID database (http://dunbrack2.fccc.edu/ProtCiD/default.aspx) with the Pfam-Pfam combination of a given DDI type and checked for available clusters and derived the number of different crystal forms as well as the minimal sequence identity. If there was a cluster existing that included the curated structure(s) of the DDI type that had a minimal sequence identity < 80% and showed at least 5 different crystal forms, we approved the cluster as supporting the DDI type. An interface present in a PDB structure supporting a specific DDI type was finally approved, if the related publication(s) described the interface to be able to mediate the respective protein-protein interaction. In case the available literature did not refer to the interface at all, we approved an interface, if the detected residue-residue contacts between the Pfam domains agreed with known stoichiometries and subcellular localizations of both proteins (i.e. interfaces between extracellular hormones and intracellular domains of receptors were not approved) and the contact between the domains provided a major interface for the interacting proteins. Furthermore, we included ProtCID information, if available, to assess whether the interface represents a crystal contact or a biologically relevant interaction. If there was a related publication available that provided evidence for the interface to be the product of crystallization or a synthetically engineered complex, we did not approve the interface. Structure determination methods as well as overall resolution for each of the 95 manually inspected PDB structures were extracted using the API of the RCSB PDB.

### *Feature annotation for machine learning*

All features for the trained classifier derived from 3did were obtained from the 3did download mentioned earlier. From 3did we used as feature the 3did score and z-score for each DDI type, the fraction of all reported structures for a given DDI type with interchain evidence as well as the total number of reported structures and the number of identified residue-residue contact pairs as defined by 3did. ProtCID cluster data for Pfam-Pfam interactions that was used as features for model training was kindly provided by the Dunbrack lab. From ProtCID we extracted for a given DDI type and corresponding ProtCID clusters the maximum number of distinct proteins in a cluster, the minimal sequence identity between proteins from different structures in the cluster, and the maximum number of crystal forms. All these ProtCID features can be indicative of how often this type of DDI was observed between diverse protein sequences that were crystallized under different conditions. The hypothesis is that the more often this DDI was found in diverse structures, the less likely it is to be an artifact of the crystallization process.

Previous studies indicated the potential use of PPI data for the identification of DDI types by scoring for an enrichment of domain pairs in interacting proteins [(Huttlin 2015, Rolland 2014)](https://www.zotero.org/google-docs/?AbQsOe). To avoid study bias, we referred to two systematically generated human protein interactome datasets either obtained from yeast two-hybrid screens (hereafter referred to as the HuRI dataset) or from affinity purification coupled to mass spectrometry experiments (hereafter referred to as the BioPlex dataset) [(Huttlin 2017, Luck 2020)](https://www.zotero.org/google-docs/?MHqM5K). We computed for HuRI and BioPlex separately for each DDI type the number of interactions between proteins with matches of the respective domain types and compared this count to counts obtained from degree-controlled randomized networks. To this end, we downloaded the HuRI and BioPlex dataset from the supplementary information from Luck et al. [(2020)](https://www.zotero.org/google-docs/?XfH7fO) and removed all homodimers prior to network randomization. 1000 degree-controlled randomized PPI networks of each of the HuRI and BioPlex network were generated using the igraph library degree_sequence() function in python. As the networks comprise only Ensembl gene IDs of the interacting protein pairs, we used Bioconductor BiomaRt to map the Ensembl gene IDs to their respective UniProt IDs. This was necessary to obtain annotations for proteins from other resources such as UniProt. We downloaded the Pfam HMM matches for all Swiss-Prot reviewed human proteins from Interpro [(Paysan-Lafosse 2023)](https://www.zotero.org/google-docs/?CkXxQG) along with their UniProt IDs in a .json file, so that we could annotate the Pfam domains for every protein in the HuRI and BioPlex networks based on their UniProt IDs. The z-scores for a given DDI type for the HuRI and BioPlex network were computed separately by first determining the number of PPIs in each respective network where both proteins matched the Pfam HMMs of a given DDI type, followed by subtracting from this count the mean number of PPIs with the DDI observed in the randomized network. Then, the difference was divided by the standard deviation of the PPI count from the randomized networks to derive the z-scores. Given the incompleteness of both networks and the restriction to human PPIs, we were not able to compute these features for 16 out of 80 curated DDI types because no proteins with matches of either of the domain types occurred in the network.

We hypothesized that AF’s ability to accurately predict the structure of a DDI might depend on the “strength” or likeliness of that DDI to be able to mediate a PPI. Using AlphaFold Multimer version 2.2.0, we generated a model for the top ranked structure of a given DDI type and computed the DockQ score between the model and actual structure to assess their similarity. We used PyMOL (version 2.5.0) to select the sequences in the structure that corresponded to both Pfam HMM matches for export. If the HMM matches did not fully cover the folded regions of either domain then the sequence(s) were extended, respectively, and an additional 10 residues added to the N- and C-terminus of the domains prior to export. This was done to ensure that all residues important for the fold of the domain were included as prior experience showed that AlphaFold models for interacting proteins are hugely misled if the folds of the domains are not complete. These sequences were used as input for model prediction by AF. For running AlphaFold, we used the default query databases provided by AlphaFold on its GitHub page. The predictions were done using the full_dbs option and the use of template was allowed by setting the flag max_template_date to 2020-05-14. We used CPUs to relax the predicted models by toggling the flag use_gpu_relax to False. Five models were predicted with a single seed per model by setting the flag num_multimer_predictions_per_model to 1. The calculation of the DockQ score was done as described by Basu & Waller [(2016)](https://www.zotero.org/google-docs/?QKQWDJ).

For the computation of the fraction of disordered residues at the interface, we used the structure of a DDI type with the highest 3did score and retrieved IUPred2A values for the protein sequence used for crystallization as well as for the full-length sequence of the proteins containing the domains forming a DDI using a local installation of IUPred2A and the short disorder prediction mode [(Mészáros, Erdős and Dosztányi 2018)](https://www.zotero.org/google-docs/?XElRSG). We retrieved the sequences used for crystallization using PyMOL (version 2.5.0) and its sequence export function. The full-length sequences were retrieved using the UniProt API. We extracted the IUPred2A scores for the residues in both proteins that were listed as contacting residues according to 3did and calculated the average IUPred2A score of those residues to use as a feature, either using the sequences used for crystallization or the full-length sequences. We used both sequence versions because sequence context can influence disorder prediction by IUPred. For assessment of statistical significance of the individual features, a Mann-Whitney-U test was performed for the approved and non-approved subsets of DDI types in the manual curation set.

# **Supplementary Figures**


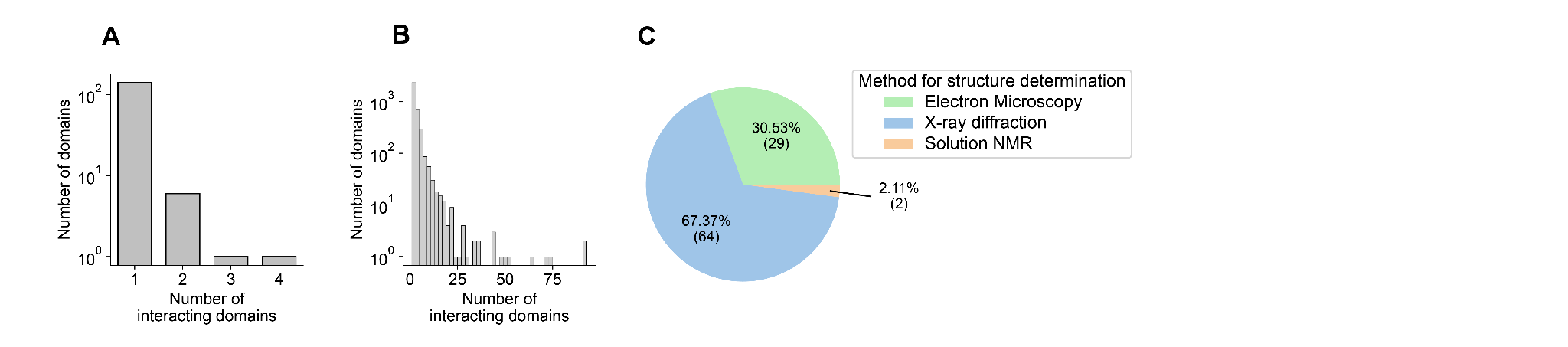


**Fig. S1.** Analysis of DDI benchmark dataset. **A**) Histogram for the number of domain types and their frequency in the DDI benchmark dataset. **B**) Histogram for the number of domain types and their frequency in all hetero-protein DDI types with interchain evidence in 3did. **C**) Pie chart showing the fractions of curated structures from DDI types in the benchmark dataset by experimental method.


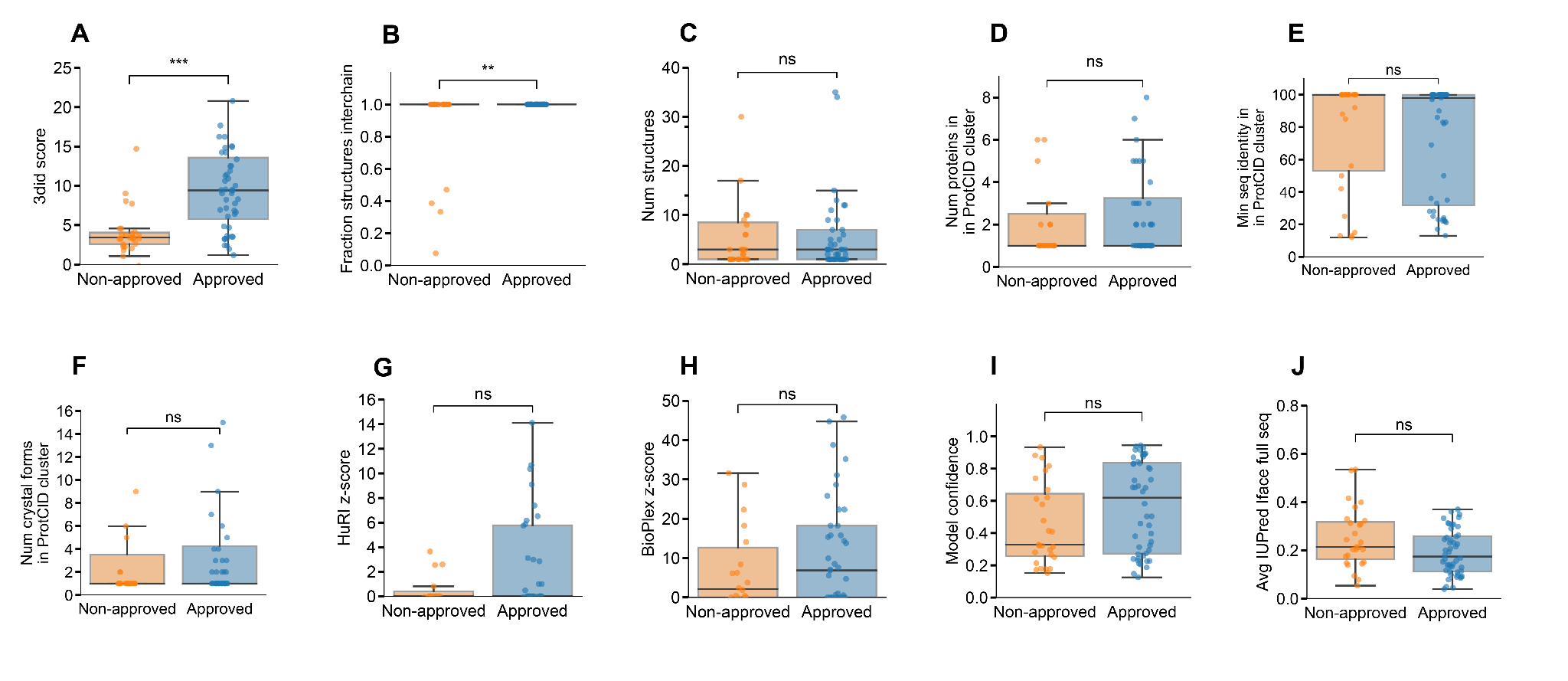


**Fig. S2.** Feature exploration to discriminate approved from non-approved DDI types. **A-J**) Boxplots showing the distribution of approved and non-approved DDI types for different features as indicated on the y-axis. Significances were computed using the Mann-Whitney-U test.


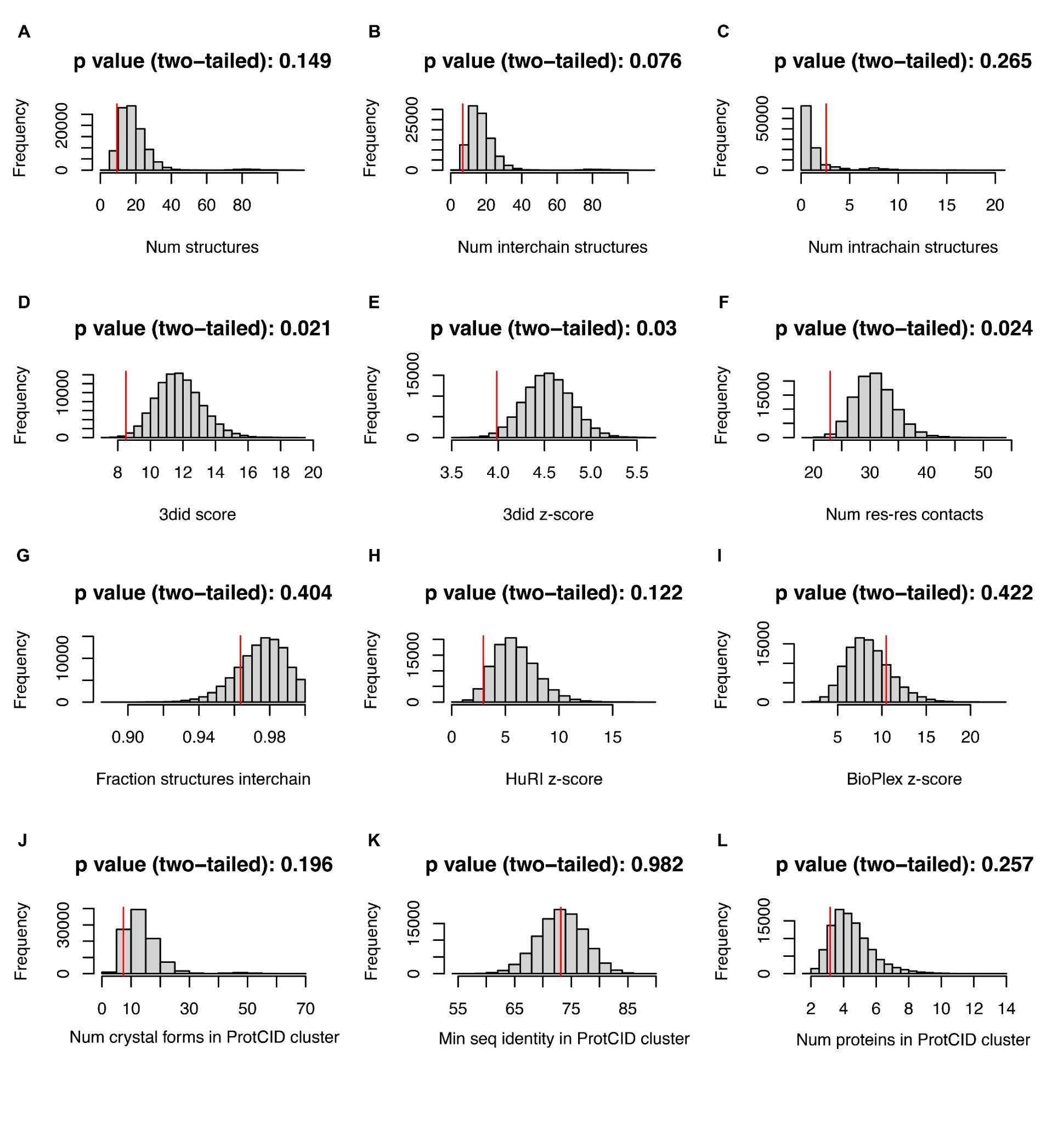


**Fig. S3.** Representativity analysis of features in manual curation DDI dataset in comparison to the subset of hetero-protein DDI types with interchain evidence. **A-L**) The average value in the different statistics for the curation dataset is shown as a red line overlaying the distribution obtained by random sampling of the 3did DDI types with n=75.


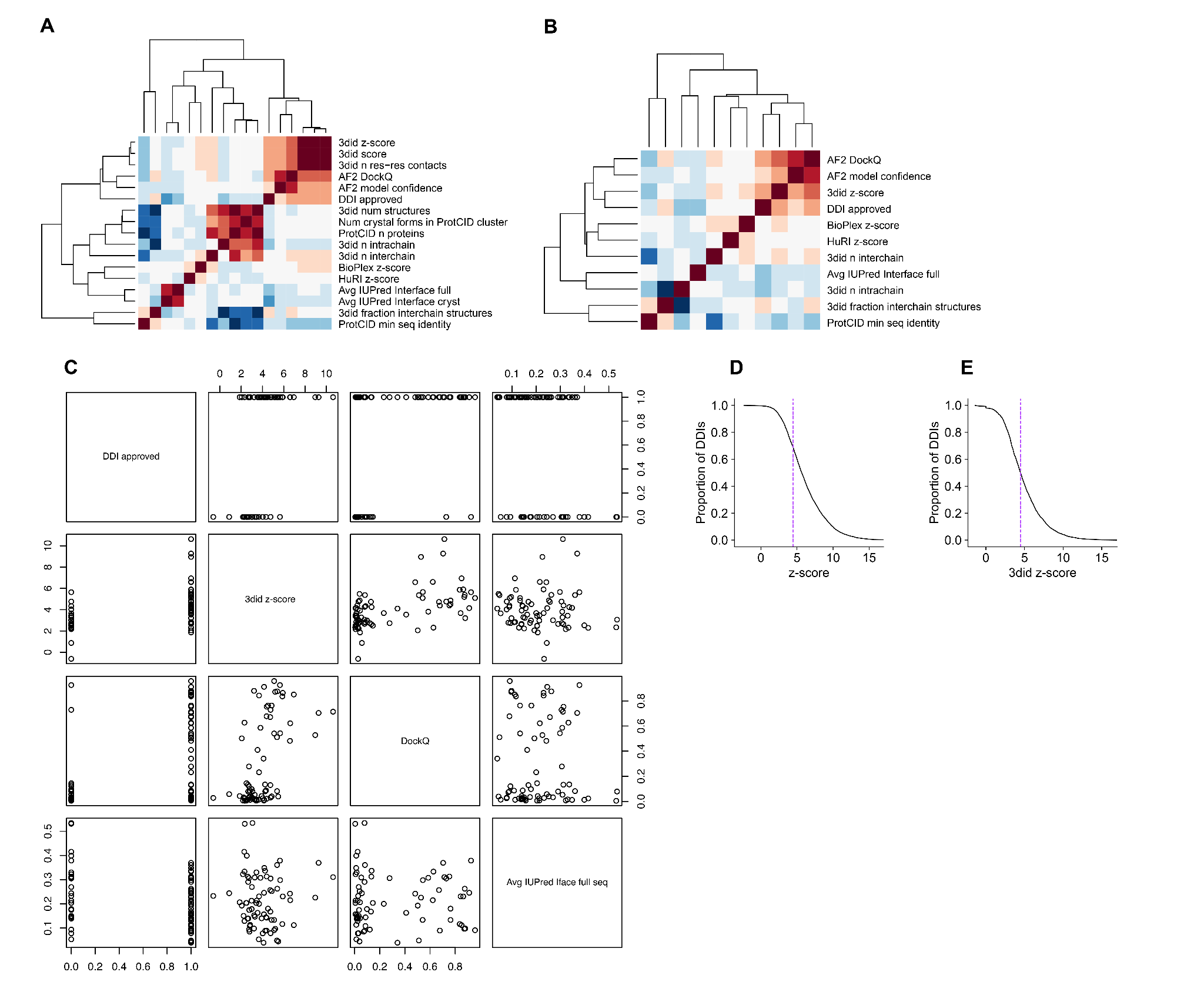


**Fig. S4.** Correlation matrices between features. **A**) Correlation matrix and hierarchical clustering of all features considered in this study. To compute correlations, pairwise complete data were used. **B**) Correlation matrix of the features used for model training. **C**) Pairwise scatterplot of the features used in the reduced model. **D-E**) Proportion of homodimeric (D) and intrachain-derived (E) DDI types in 3did retained based on increasing 3did z-score cutoffs. The horizontal line indicates the 4.47 3did z-score cutoff suggested from training on heteroprotein DDI types with interchain evidence.

**
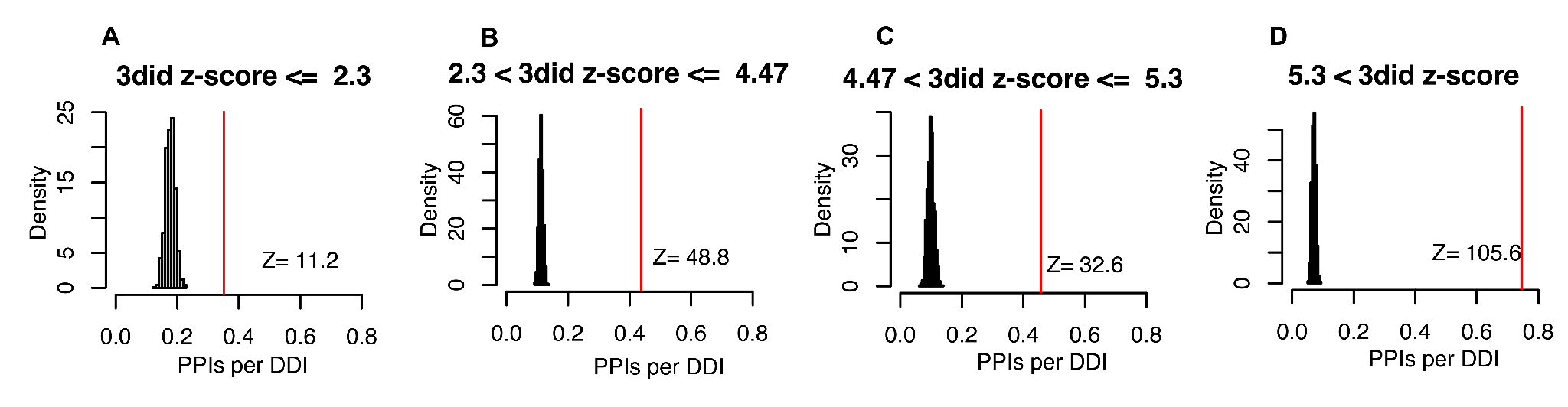
**

**Fig. S5.** Enrichment analysis for the number of PPIs in HuRI predicted with a DDI using different subsets of DDI types based on increasing confidence as defined by indicated 3did z-score ranges (**A-D**). Z-scores indicated in the plot represent enrichment over background distribution. The number of PPIs with a predicted DDI were normalized by the number of DDI types available in the corresponding subset.


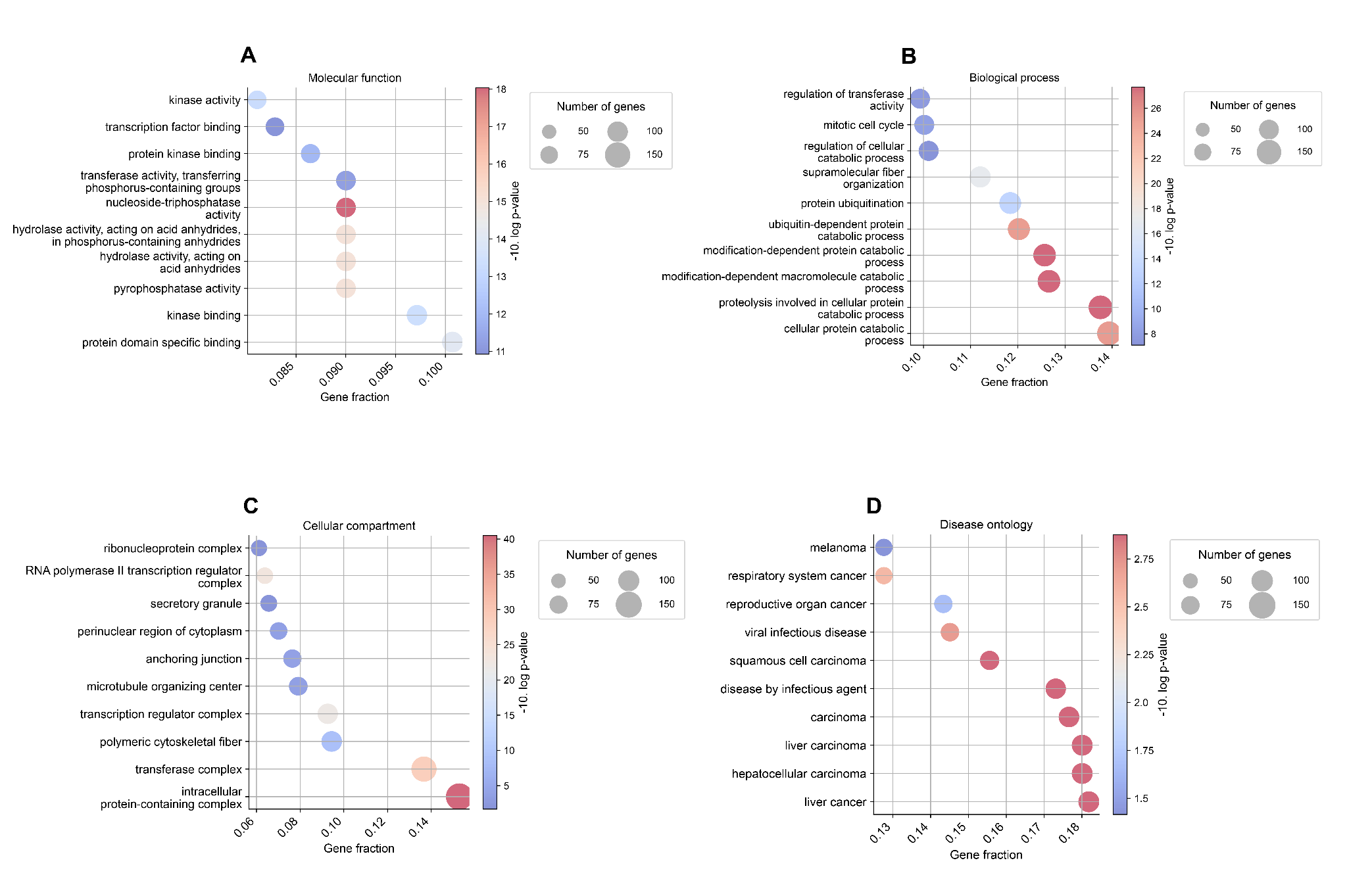


**Fig. S6.** GO enrichment analysis for four categories (molecular function, biological process, cellular compartment and disease ontology, A-D) performed on proteins in PPIs from HuRI for which a DDI was predicted. All proteins in the HuRI dataset were used as background.


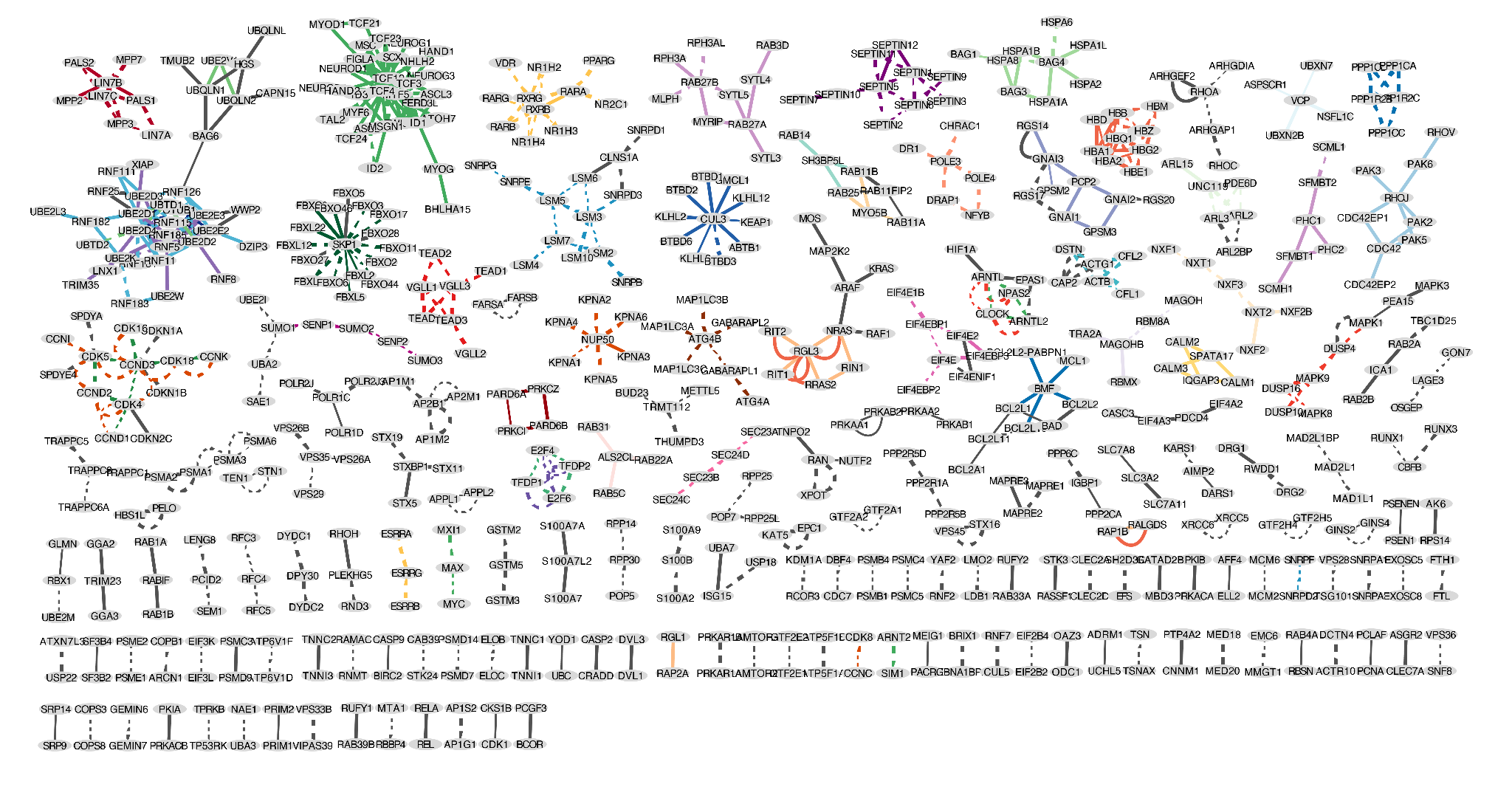


**Fig. S7.** Network of PPIs from HuRI with predicted and successfully structurally modeled DDIs. Edges are colored by DDI types. Nodes are labeled with gene symbols. Thin and thick edges correspond to those for which a resolved or no structure is available, respectively. Dashed and solid lines indicate PPIs for which a high confident AF model is or is not available from using full length protein sequences as reported in [Burke et al. (2023)](https://www.zotero.org/google-docs/?IpOZFW).

**References**

[Basu, Sankar, and Wallner, Björn, ‘DockQ: A Quality Measure for Protein-Protein Docking Models’, *PloS One*, 11/8 (2016), e0161879](https://www.zotero.org/google-docs/?RA816S)

[Burke, David F., Bryant, Patrick, Barrio-Hernandez, Inigo, Memon, Danish, Pozzati, Gabriele, Shenoy, Aditi, et al., ‘Towards a Structurally Resolved Human Protein Interaction Network’, *Nature Structural & Molecular Biology*, 30/2 (2023), 216–25](https://www.zotero.org/google-docs/?RA816S)

[Huttlin, Edward L., Bruckner, Raphael J., Paulo, Joao A., Cannon, Joe R., Ting, Lily, Baltier, Kurt, et al., ‘Architecture of the Human Interactome Defines Protein Communities and Disease Networks’, *Nature*, 545/7655 (2017), 505–9](https://www.zotero.org/google-docs/?RA816S)

[Huttlin, Edward L., Ting, Lily, Bruckner, Raphael J., Gebreab, Fana, Gygi, Melanie P., Szpyt, John, et al., ‘The BioPlex Network: A Systematic Exploration of the Human Interactome’, *Cell*, 162/2 (2015), 425–40](https://www.zotero.org/google-docs/?RA816S)

[Luck, Katja, Kim, Dae-Kyum, Lambourne, Luke, Spirohn, Kerstin, Begg, Bridget E., Bian, Wenting, et al., ‘A Reference Map of the Human Binary Protein Interactome’, *Nature*, 580/7803 (2020), 402–8](https://www.zotero.org/google-docs/?RA816S)

[Mészáros, Bálint, Erdős, Gábor, and Dosztányi, Zsuzsanna, ‘IUPred2A: Context-Dependent Prediction of Protein Disorder as a Function of Redox State and Protein Binding’, *Nucleic Acids Research*, 46/W1 (2018), W329–37](https://www.zotero.org/google-docs/?RA816S)

[Paysan-Lafosse, Typhaine, Blum, Matthias, Chuguransky, Sara, Grego, Tiago, Pinto, Beatriz Lázaro, Salazar, Gustavo A, et al., ‘InterPro in 2022’, *Nucleic Acids Research*, 51/D1 (2023), D418–27](https://www.zotero.org/google-docs/?RA816S)

[Rolland, Thomas, Taşan, Murat, Charloteaux, Benoit, Pevzner, Samuel J., Zhong, Quan, Sahni, Nidhi, et al., ‘A Proteome-Scale Map of the Human Interactome Network’, *Cell*, 159/5 (2014), 1212–26](https://www.zotero.org/google-docs/?RA816S)
